# Spontaneous Detorsion of Sigmoid Volvulus in a patient with Nineteen-Volvulus episode history: A rare outcome of an extremely rare clinical entity

**DOI:** 10.12669/pjms.37.7.4703

**Published:** 2021

**Authors:** Sabri Selcuk Atamanalp, Esra Disci, Cansu Tatar Atamanalp, Refik Selim Atamanalp

**Affiliations:** 1 Prof. Sabri Selcuk Atamanalp, MD. Department of General Surgery, Faculty of Medicine, Ataturk University, Erzurum, Turkey; 2 Esra Disci, MD. Assistant Professor, Department of General Surgery, Faculty of Medicine, Ataturk University, Erzurum, Turkey; 3 Cansu Tatar Atamanalp, MD. Assistant, Department of Pediatrics, Haseki Education and Research Hospital, Istanbul, Turkey; 4 Refik Selim Atamanalp, MD. Assistant, Department of Pathology, Prof. Dr. Cemil Tascioglu City Hospital, Istanbul, Turkey

**Keywords:** Sigmoid volvulus, Recurrence, Spontaneous detorsion

## Abstract

Sigmoid volvulus (SV) recurrence more than 10 times is an extremely rare clinical entity and spontaneous detorsion is a rare outcome of SV. In this paper, we report a case with 19 previous SV attacks, in last of which spontaneous detorsion occurred. Such a multiple-episode history as well as an unexpected recovery was unique in a 1,036-case clinical profession of Ataturk University with SV over a 54.5-year period.

## INTRODUCTION

Sigmoid volvulus (SV), a closed-loop colonic obstruction, is a rare disease worldwide, while it is relatively common in some Asian, African, South American, and Eastern European countries.^1^ Although the recurrence of SV is not a mystery, multiple recurrence attacks, particularly more than 10 times, are extremely rare with a few cases reported to date.^2^ Spontaneous detorsion is an unusual outcome of SV, which is seen in fewer than 2% of SV cases.^3^ We report herein a case of a 72-year-old man with 19-recurrence attack history, the last of which healed spontaneously. We encountered both of these clinical characteristics for the first time in a 1,036-case clinical experience with SV over a 54.5-year period (from June 1966 to January 2021) in Ataturk University.

## CASE REPORT

**Fig.1:**
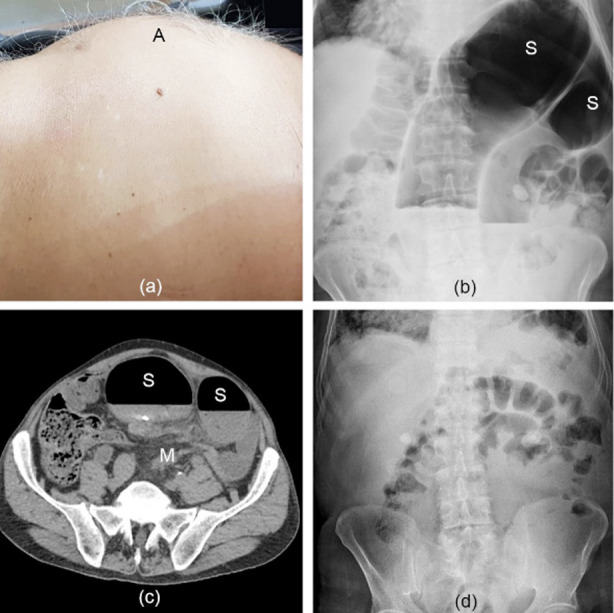
(a) Clinical appearance (A: asymmetrically distended abdomen), (b) Plain abdominal X-ray image (S: twisted sigmoid colon segments forming coffee bean sign), (c) Axial abdominal CT image (S: dilated sigmoid colon, M: mesenteric whirl sign), (d) Control plain abdominal X-ray image.

A 72-year-old man presented with abdominal pain, distention, and obstipation for 12 hours. He was previously diagnosed with coarctation of aorta and coronary disease. In anamnesis, a history of 19 volvulus attacks was present. All SV episodes including the first one in childhood, had been decompressed endoscopically. Physical examination revealed asymmetrical abdominal distention more prominent in left upper quadrant (Fig.1a), abdominal tenderness, hyperkinetic bowel sound, and an empty rectum. Abdominal X-ray radiography demonstrated dilated sigmoid colon segments forming coffee bean sign (Fig.1b). Abdominal CT scan presented dilated sigmoid colon segments in addition to mesenteric whirl sign (Fig.1c). According to the clinical and radiological findings, SV with nongangrenous bowel was diagnosed. Following resuscitation, an endoscopic detorsion was planned. During the preparation period, the patient defecated normal-appearing stool together with abundant degassification. A follow-up examination revealed a relaxed abdomen with minimal tenderness. There was no abnormal finding in the control abdominal X-ray radiography (Fig.1d). Due to the poor general condition of the patient (American Society of Anesthesiologists-ASA score 4) arising from serious comorbidities, an elective surgery was not suggested, and he didn’t approve percutaneous endoscopic colopexy (PEC). The patient was discharged after a 24-hour observation.

## DISCUSSION

SV tends to recur in about one fourth of all cases. The recurrence rate principally depends on the treatment method, and it is more common in patients treated with endoscopic or surgical detorsion alone. Similarly, pediatric SV is considerably disposed to recurrence.^4^ On the other hand, due to the elective surgical treatment of some selected patients in addition to the loss of some others during the observation period, multiple recurrence attacks more than 10 times are extremely rare with a few cases reported to date.^2^ On SV, we have a 1,036-case experience, which is the largest single-center SV series in the world.^5^ Of our 935 patients, whose records were available, 242 (25.9%) presented a previous volvulus episode with a mean 1.4 times. In our series, 206 patients had a single attack, 31 patients had 2-5 attacks, two patients had 6-10 attacks, and three patients had 11 or more attacks, while the presented case was the first patient with 19 attacks.

The sigmoid colon occasionally twists and untwists, and these are accepted physiologically. Torsions less than 180° are generally asymptomatic and result in spontaneous untwisting. If the torsion exceeds 180°, intestinal passage is blocked and complications including bowel ischemia, necrosis, and perforation may occur.^6^,^7^ In such patients, spontaneous detorsion is difficult or even impossible due to gas generation in the closed loop and it is expected in fewer than 2% of the cases.^1^ The presented case was the first patient who recovered spontaneously following the diagnosis of SV during the hospitalization period.

It is clear that every volvulus attack carries some risks of mortality and morbidity.^5^ However, in our opinion and experience, this disadvantage may provide some other interesting advantages in patients with multiple volvulus attacks. Firstly, due to their previous clinical experiences, such patients generally get used to the clinical presentation of SV. Thus, they may visit to hospital without loss of time. Secondly, SV diagnosis is frequently made by patients at admission, which may provide early and correct diagnosis. Finally, repetitive attacks facilitate not only twisting but also untwisting of the sigmoid colon, which may lead to spontaneous detorsion of SV.

Regarding the treatment options in patients with poor general conditions, like the presented case, in our opinion and experience, observation is generally preferred by both physicians and the patients to avoid unintended consequences. Neither emergency nor elective surgical sigmoid colectomy is thought as the optimal choice due to their poor prognosis, which consists of 10-30% of mortality and 20-40% of morbidity rates.^5^ Instead, emergency or preferably elective PEC may be a proper option. Despite the limited experience on PEC treatment in SV including fewer than 100 cases reported to date, it is a hope in selected patients.^8^,^9^ PEC doesn’t require general anesthesia and may be applied under endoscopic premedication together with local anesthesia, and may demonstrate acceptable results with 5-13% of mortality and 13-18% of morbidity rates.^10^

### Authors Contribution:

**SSA:** Data collection, manuscript writing.

**ED:** Data collection, revision of the final draft.

**CTA:** Revision of the final draft.

**RSA:** Revision of the final draft.

**SSA**: Is responsible for responsible and accountable for the accuracy or integrity of the work.
